# *Treponema pallidum* subsp. *pallidum* strains DAL-1 and Philadelphia 1 differ in generation times *in vitro* as well as during experimental rabbit infection

**DOI:** 10.1371/journal.pone.0304033

**Published:** 2024-05-24

**Authors:** Juraj Bosák, Lenka Mikalová, Matěj Hrala, Petra Pospíšilová, Martin Faldyna, David Šmajs

**Affiliations:** 1 Department of Biology, Faculty of Medicine, Masaryk University, Brno, Czech Republic; 2 Department of Infectious Diseases and Preventive Medicine, Veterinary Research Institute, Brno, Czech Republic; GGD Amsterdam, NETHERLANDS

## Abstract

In this work, we determined that *Treponema pallidum* subsp. *pallidum* (TPA) DAL-1 (belonging to Nichols-like group of TPA strains) grew 1.53 (± 0.08) times faster compared to TPA Philadelphia 1 (SS14-like group) during *in vitro* cultivations. In longitudinal individual propagation in rabbit testes (n = 12, each TPA strain), infection with DAL-1 manifested clinical symptoms (induration, swelling, and erythema of testes) sooner than Philadelphia 1 infection, which resulted in a significantly shorter period of the experimental passages for DAL-1 (median = 15.0 and 23.5 days, respectively; p < 0.01). To minimize the confounding conditions during rabbit experiments, the growth characteristics of DAL-1 and Philadelphia 1 strains were determined during TPA co-infection of rabbit testes (n = 20, including controls). During two weeks of intratesticular co-infection, DAL-1 overgrew Philadelphia 1 in all twelve testes, regardless of inoculation ratio and dose (median of relative excess DAL-1 multiplication = 84.85×). Moreover, higher DAL-1 to Philadelphia 1 inoculum ratios appeared to increase differences in growth rates, suggesting direct competition between strains for available nutrients during co-infection. These experiments indicate important physiological differences between the two TPA strains and suggest growth differences between Nichols-like and SS14-like strains that are potentially linked to their virulence and pathogenicity.

## Introduction

*Treponema pallidum* subsp. *pallidum* (TPA) is the causative agent of syphilis, a sexually transmitted disease with increasing incidence in many areas of the world, including the USA, China, and Europe [1, 2].

Experimental studies in rabbits have historically shown biological differences among syphilis-causing strains. For instance, the characteristics of testicular and skin lesions in rabbits, as well as the frequency of periorchitis in Mexico A strain infections, differed from those of the Nichols and Chicago strains [3]. In another study, rabbit propagation of the CDC-SF003 and CDC-SF007 TPA strains resulted in negligible orchitis and late onset of seroreactivity [4]. However, none of these observations have been correlated with differences in genomic sequences.

Later, genetic analyses of TPA reference strains and human clinical isolates revealed two genetically distinct groups of TPA strains, one related to the reference strain Nichols (Nichols-like strains) and the other to the reference strain SS14 (SS14-like strains) [5–9]. While the above-mentioned Mexico A, CDC-SF003, and CDC-SF007 strains belong to the SS14-like group, the Nichols and Chicago strains belong to the Nichols-like group.

After decades of unsuccessful attempts to continuously cultivate TPA *in vitro*, a long-term logarithmic multiplication of TPA in a rabbit epithelial cell co-incubation system has been introduced [10]. Using this technique, differences in the growth rates (i.e., in the length of generation times) among Nichols, Mexico A, and SS14 strains were found [11], which suggests important differences in the physiology of syphilis treponemes.

TPA strain DAL-1 was isolated in 1991 (Dallas, Texas, USA) from a 21-year-old African American woman [12]. Strains DAL-1 and Nichols differ in the sequence of the TP0136 gene, in the number of repetitions in the TP0470 gene, and in an additional 38 nt changes [13]. TPA Philadelphia 1 was isolated in 1988 (Philadelphia, Pennsylvania, USA) and characterized later [14]. Similarly, Philadelphia 1 differs from the SS14 strain in 14 nucleotide positions [15]. Based on the comparison of Nichols and SS14 genomes [6], DAL-1 and Philadelphia 1 strains differ at more than 600 nucleotide positions. Compared to DAL-1, Philadelphia 1 strain is more related to contemporary TPA strains (SS14-Omega clade) [16].

This study compared the growth characteristics of TPA DAL-1 (Nichols-like cluster) and Philadelphia 1 (SS14-like cluster) under *in vitro* conditions and during experimental rabbit infection. To maintain identical conditions in the animal model, experimental co-infection with both TPA strains was performed.

## Materials and methods

### Source of *T*. *pallidum* strains

Both *T*. *pallidum* strains (DAL-1 and Philadelphia 1) were kindly provided by Dr. D. L. Cox (Centers for Disease Control and Prevention, Atlanta) as frozen suspensions from rabbit testes with unknown concentrations of treponemal cells.

### *In vitro* cultivation of *T*. *pallidum* strains

Both *T*. *pallidum* strains (DAL-1 and Philadelphia 1) were successfully cultivated *in vitro* over one year using a previously published cultivation system [10]. Briefly, treponemes were cultivated in the presence of TpCM-2 medium (4 ml), Sf1EP rabbit cells (50,000 cells), and a low oxygen atmosphere using a 6-well plate format. Compared to the original protocol, the O_2_ level was raised from 1.5% to 2.5%, and equilibration in a brewer jar was not used. Every seven days, treponemes were detached using Trypsin/EDTA (2 × 500 μl, 37 ⁰C, 5 min) and briefly centrifuged (150 × g, 5 min) to deplete rabbit cells. A supernatant containing treponemes (250 μl) was directly subcultured in a new well containing fresh TpCM-2 medium and Sf1EP cells. As a result, the treponemal population was diluted by a factor of 20 (i.e., 250 μl out of 5 ml of collected culture) during each passage.

For growth quantification, long-term *in vitro* cultures were used as the source of treponemes, and 7-day cultures were adjusted for the defined inoculation dose of treponemes (250,000 bacterial cells). Each biological experiment (n = 4) was performed in four technical replicates using a 24-well plate format. Here, treponemes (250,000 cells based on dark-field microscopy) were co-cultivated with rabbit Sf1EP (10,000 cells) in TpCM-2 medium (final volume of 1.5 ml) for seven days in a low-oxygen atmosphere (2.5%). Then, treponemes were collected using Trypsin/EDTA (2 × 250 μl), which resulted in a 2-ml final volume of TPA cultures. Cell suspensions with living treponemes (5 μl) were immediately subjected to dark-field microscopy, and another aliquot (200 μl) was frozen (-20°C) for qPCR analysis.

For the *in vitro* co-cultivation experiment, both TPA strains were inoculated (i.e., 250,000 cells each, estimated from DFM) to the same well (n = 6) and cultivated for two seven-day subcultures as described above.

### Dark-field microscopy (DFM) for the detection of treponemes

Fresh treponemal cultures (5 μl) were subjected to dark-field microscopy, and treponemes were counted using a BX53 microscope (Olympus Corporation, Tokyo, Japan) at 400× magnification. Five individual fields were analyzed for each sample, and the number of treponemes was calculated. Based on the size of the visual field and sample volume under the coverslip, one treponeme per field represented 340,000 treponemes per ml of culture. In the case of low treponemal numbers (i.e., < 1 TPA per field), fifty fields of specimen were screened. The DFM detection limit was calculated to be 6,800 treponemes per ml.

### Quantitative real-time PCR (qPCR) for the detection of treponemes

For quantification of TPA strains during single *in vitro* cultivation, qPCR detection was designed for the *pol*A gene (TP0105; product size 129 bp) using two primers (5’–GAGTGTGCAGTCCGCTATGC–3’ and 5’–AGGCAAAAGCGGCATTTCTA–3’) and one probe qPCR_polA_probe (5’–FAM-TCCGCTTGGAAACAGCAGGATTG-BHQ–3’) [17]. qPCR was performed using the Azure Cielo Real-Time PCR System (Azure Biosystems). Each PCR reaction (20 μl) contained Luna Universal Probe qPCR Master Mix (10 μl, New England Biolabs), primers (0.08 μl each, final conc. 400 nM), FAM-labeled probe (0.04 μl, final conc. 200 nM), template DNA (i.e., 5 μl of treponemal *in vitro* culture), and nuclease-free water (4.8 μl). PCR cycling conditions were 95°C (10 min), followed by 40 cycles at 95°C (10 s), and 60°C (30 s). A standard curve for TPA DNA was constructed using 10-fold serial dilutions (i.e., 10^0^–10^6^ copies/μl) of the pCR2.1-TOPO vector (Invitrogen) containing the cloned *pol*A fragment.

### Animal model for experimental *T*. *pallidum* infection

All animals in this study were male New Zealand White rabbits (8–12 weeks old) with negative RPR serology (Omega Diagnostics). Rabbits were housed individually, under controlled conditions, in wire mesh cages in the animal care facility at the Veterinary Research Institute (Brno, Czech Republic). The animals were housed and handled under conditions set by the Branch Commission for Animal Welfare of the Ministry of Agriculture of the Czech Republic.

Rabbits were inoculated in both testes with a defined number of treponemes assessed using DFM. Symptom onset (i.e., redness, swelling, induration of testes) was monitored daily. The rapid plasma reagin (RPR) test was done on selected individuals to monitor the elevation of antibodies and support the observed clinical symptoms of treponemal infection. Animals were euthanized under general anesthesia using medetomidine IM (0.2 mg/kg, Cepetor), ketamine IM (15 mg/kg, Narkamon), and T-61 IV (3 ml, Intervet International). The testicles were aseptically removed, sliced, and placed in sterile PBS (3–5 ml). Treponemes were released by tissue agitation (100 cycles/min for 25 min) as described by Lukehart and Marra [18].

### Individual propagation of DAL-1 and Philadelphia 1 in laboratory rabbits

In this study, thirteen laboratory rabbits were experimentally infected with *T*. *pallidum* strain DAL-1 (n = 7) or strain Philadelphia 1 (n = 6) and used to propagate treponemal strains *in vivo*. Detailed information about animal handling and isolation of treponemes was done as described above. S1 Table contains information about individual rabbit infections, including inoculum (i.e., a dose of treponemes), the day of symptoms onset (i.e., redness, swelling, induration of testes), RPR test results, and the number of treponemes extracted from each testis. In total, 12 testes were inoculated with each TPA strain. The right testis of two rabbits (S1 Table) were not used for DAL-1 inoculations and served as negative controls.

### The intratesticular co-infection of DAL-1 and Philadelphia 1 in laboratory rabbits

**Fig 1:**
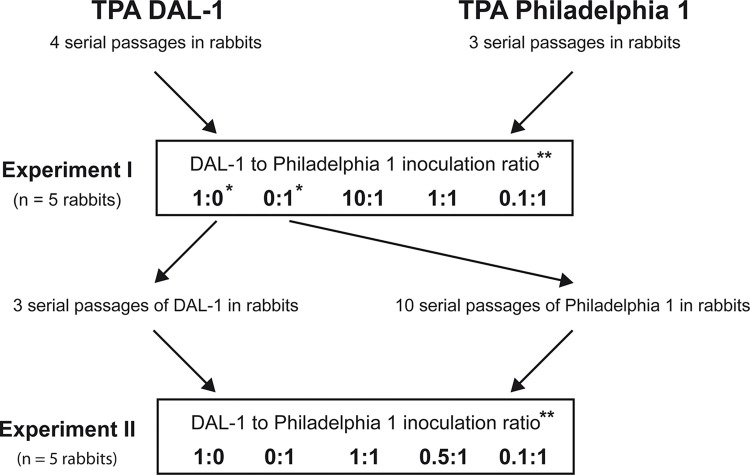
Scheme of two intratesticular co-cultivation experiments of DAL-1 and Philadelphia 1 strains in rabbits. Before each experiment, several serial passages of individual TPA strains were performed to ensure that both strains were well adapted for rabbit infection. In each experiment, two rabbits were infected with only one TPA strain (i.e., controls), and three rabbits were infected with a mixture of both TPA strains in different ratios. After 14 days of experimental infection, testicular samples were collected and frozen for qPCR analysis of treponemal yield. *Two rabbits infected with individual TPA strains in Experiment I were used as a source of treponemes for Experiment II. **Please note that the inoculation dose was initially estimated using DFM, and after the experiments, it was refined using qPCR quantification.

The intratesticular co-infection of treponemal strains in laboratory rabbits (n = 10) was performed in two independent and time-separated experiments (Experiment I and II) (Fig 1). Before each experiment, DAL-1 and Philadelphia 1 strains were individually propagated in laboratory rabbits (3–10 passages) to ensure that both strains were well adapted to rabbit infection. On the day of inoculation, two rabbits (one for each TPA strain) were used as a source of living treponemes, which were extracted as described above. No more than one hour after euthanasia, treponeme numbers and motility (viability) were assessed using DFM, and treponemes in PBS were inoculated into both testes (500 μl per testis) of a naive experimental rabbit. The exact numbers of inoculated treponemes were calculated later using qPCR (see S2 Table). In both experiments, two rabbits were inoculated with only one strain (DAL-1 or Philadelphia 1), while three rabbits were infected using a mixture of both TPA strains but in different ratios (Fig 1).

After 14 days of treponemal infection, experimental animals were euthanized. The testicles were aseptically removed, sliced, and placed in a sterile PBS (5 ml and 3 ml in Experiments I and II, respectively). Treponemes were released (see above) and were frozen at −80°C. Aliquots of the mixture (200 μl) were used for qPCR analyses.

### Strain-specific qPCR detection of DAL-1 and Philadelphia 1

To measure multiplication of individual TPA strains in co-cultures and co-infections, the sequence difference in gene TP0136 (encoding the fibronectin-binding protein [19]) between DAL-1 (CP003115, [13]) and Philadelphia 1 (CP035193, [15]) was used to design a strains specific qPCR detection.

Primers and probe for qPCR were designed to specifically amplify the TP0136 gene (S3 Table). The forward primer (5’–GCGAATACTTGCTCATCGGCG–3’) was the same for both TPA strains, while the reverse primers were specific for DAL-1 and Philadelphia 1 (5’–CACAATATCCTGCCTTCCACTTCGTAGG–3’ and 5’–GCTCTCTTTCAATCGCATGCAG–3’, respectively). The resulting qPCR product size was 101 bp for Philadelphia 1 and 120 bp for DAL-1. Probe MAD-probe-FAM (5’–FAM-GGGGCTACGGGGAAATAAAGCTGG-BHQ–3’) was used to recognize both treponemal PCR products. The detection of individual TPA strains was performed in separate wells using a QuantStudio^TM^ 3 Real-Time PCR system (Applied Biosystems). Each PCR reaction volume (20 μl) contained QuantiFast Probe PCR Master Mix (10 μl, QIAGEN), primers (0.08 μl for each, final conc. 400 nM), FAM-labeled probe (0.04 μl, final conc. 200 nM), DNA (1–5 μl, see below), and nuclease-free water. PCR cycling conditions were 95°C (3 min), followed by 40 cycles at 95°C (10 s), and 60°C (30 s). A standard curve was constructed using 10-fold serial dilutions (10^0^–10^6^ copies/μl) of the pCR2.1-TOPO vector containing both versions of the cloned TP0136 products.

For qPCR detection, DNA was isolated from treponemal mixtures (200 μl) using QIAamp DNA Blood Mini kit (QIAGEN) according to the Blood and Body Fluids protocol. DNA was eluted into AE buffer (100 μl).

### Statistical analysis

A two-tailed Student’s t-test was used for statistical analysis. P-values lower than 0.05 were considered statistically significant and are denoted with asterisks according to statistical significance (*p < 0.05; **p < 0.01; and ***p < 0.001). GraphPad Prism 10 software was used for calculations.

## Results

### *In vitro* growth of *T*. *pallidum* strains DAL-1 and Philadelphia 1

**Fig 2:**
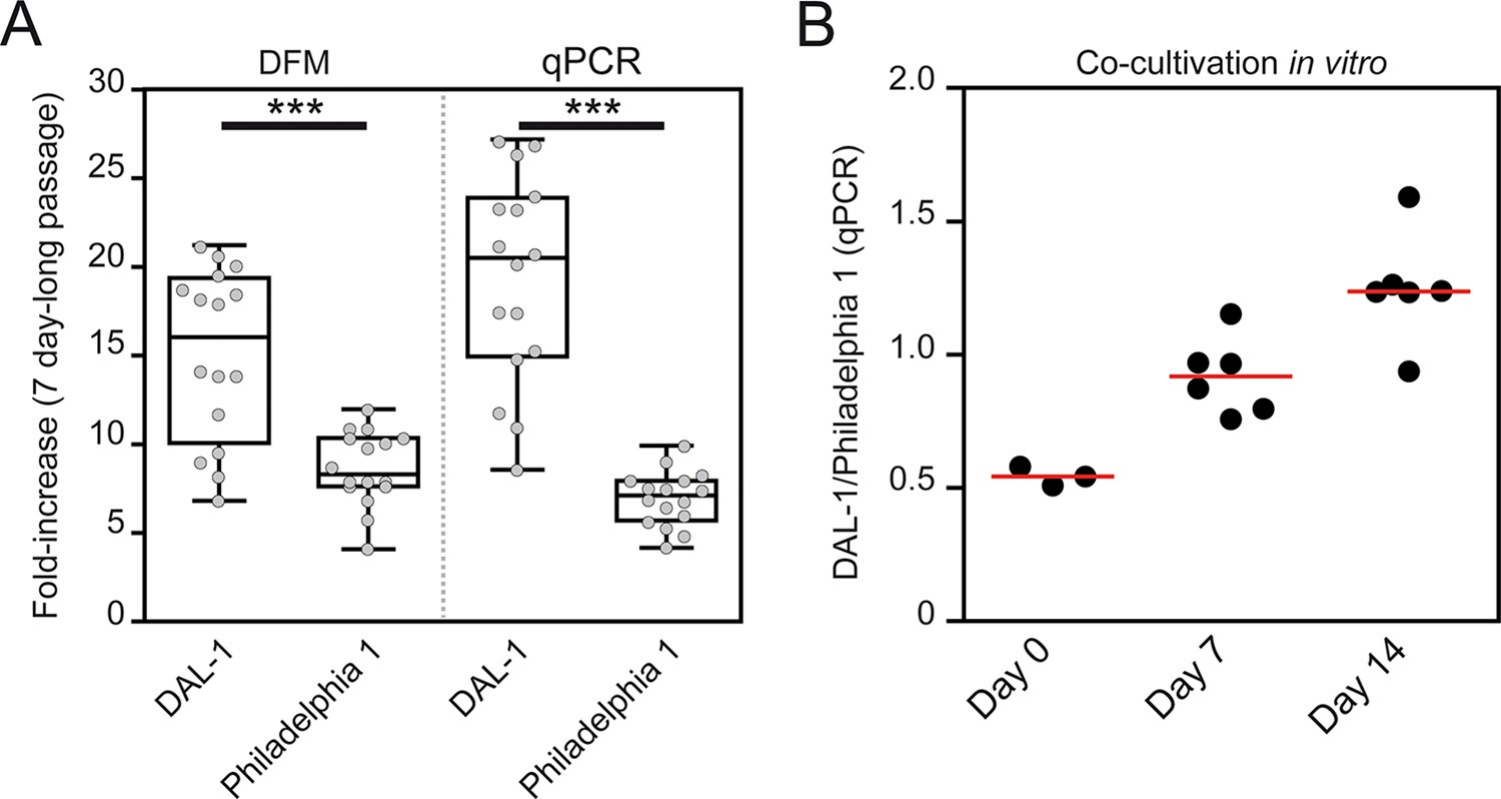
*In vitro* multiplication of TPA DAL-1 and TPA Philadelphia 1. **(A)** A fold increase of DAL-1 and Philadelphia 1 individually cultivated in 16 replicates (four biological replicates with four technical replicates; grey dots) as revealed by dark-field microscopy (left) and quantitative real-time PCR (right). The Box and Whisker graphs represent the median and 95% confidence interval. Unpaired Student’s t-test was used to calculate statistical significance (***p < 0.001). Please note that the TPA count from DFM (i.e., 250,000 treponemes) was used to inoculate individual experimental wells. For qPCR quantification of treponemal growth, inoculum doses were also quantified by qPCR, and these values were used for calculation. **(B)** Both TPA strains were co-cultivated *in vitro*. The DAL-1 to Philadelphia 1 ratio was quantified at three time points (i.e., inoculation, 1^st^ passage, and 2^nd^ passage) using strain-specific qPCR detection of the TP0136 gene. Data from individual wells are shown (black circles, average of two qPCR replicates; red bar, median). Complete *in vitro* data are shown in S4 Table.

The DAL-1 strain grew significantly faster than the Philadelphia 1 strain *in vitro* (p < 0.001, Fig 2A). In the 7-day subculture, the average fold increase was 15.13 (3.84 generations) for DAL-1 and 8.65 (3.07 generations) for Philadelphia 1. In accordance with Edmondson et al. [10], the maximal fold increase was also calculated from the five highest values to minimize the effect of suboptimal *in vitro* conditions in certain wells. The maximal fold increase during cultivation was found to be 20.07 and 10.88 (equivalent to 4.33 and 3.44 generations) for DAL-1 and Philadelphia 1, respectively. DFM results were further confirmed by qPCR detection (i.e., TP0105 detection), which showed a maximal fold increase of 25.55 and 8.60 (equivalent to 4.68 and 3.11 generations) for DAL-1 and Philadelphia 1, respectively. Both quantifications clearly demonstrated faster growth of DAL-1 compared to Philadelphia 1.

The faster growth of the DAL-1 strain was also found during co-cultivation of both strains *in vitro* (Fig 2B). Using strain-specific qPCR quantification (i.e., detection of TP0136 gene), DAL-1/Philadelphia 1 ratio changed from 0.544 to 0.919 and to 1.250 after 7 and 14 days of cultivation, respectively. In co-cultivation, the DAL-1 strain grew 1.53 (± 0.080) times faster than the Philadelphia 1 strain, which was close to the growth rate differences observed in single *in vitro* cultures.

### Growth of *T*. *pallidum* DAL-1 and Philadelphia 1 during individual infections in rabbits

**Fig 3:**
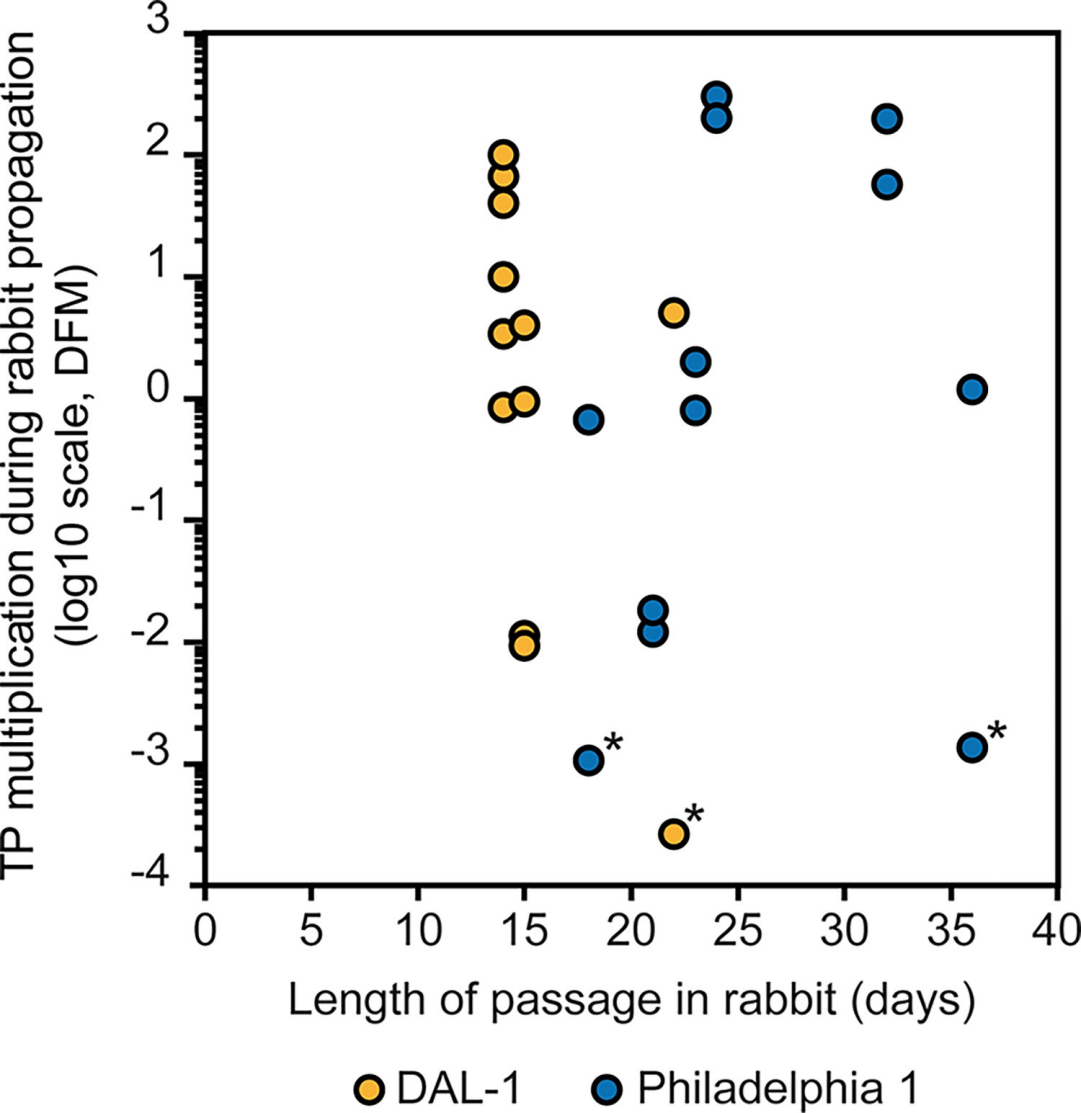
Multiplication of TPA DAL-1 (n = 12) and Philadelphia 1 (n = 12) propagated in rabbits. The multiplication of treponemes (defined as the ratio between the number of treponemes per ml at the beginning and the end of the passage) is shown on a log10 scale. The length of passages in rabbits was derived based on the clinical manifestation of symptoms. The asterisks indicate that treponemes were present in numbers lower than the DFM detection limit (i.e., 50 fields of view were analyzed). In these cases, the DFM detection limit value (i.e., 6.8 × 10^3^ per ml) was used to calculate treponemal multiplication.

During longitudinal experimental rabbit passages in 12 individual testes for each TPA strain (S1 Table), DAL-1 and Philadelphia 1 infections showed distinct manifestation despite similar inoculation doses (0.51–25.5 × 10^6^ and 0.21–6.38 × 10^6^, respectively; p = 0.1195). Rabbit testicular infection with DAL-1 manifested clinical symptoms (induration, swelling, and erythema of testes) sooner than Philadelphia 1 infection, which resulted in a significantly shorter experimental passage for DAL-1 (median number of days = 15.0 and 23.5, respectively; p < 0.01). However, the DFM results of individual passages expressed as a multiplication of the inoculation number were highly variable for both the DAL-1 and Philadelphia 1 (Fig 3). In three cases (one for DAL-1 and two for Philadelphia 1), the same inoculum resulted in an undetectable number of treponemes in one testis and an increased number of treponemes in the second testis of the same animal at the end of the passage (S1 Table).

### Growth of *T*. *pallidum* DAL-1 and Philadelphia 1 during intratesticular co-infection in rabbits

**Fig 4:**
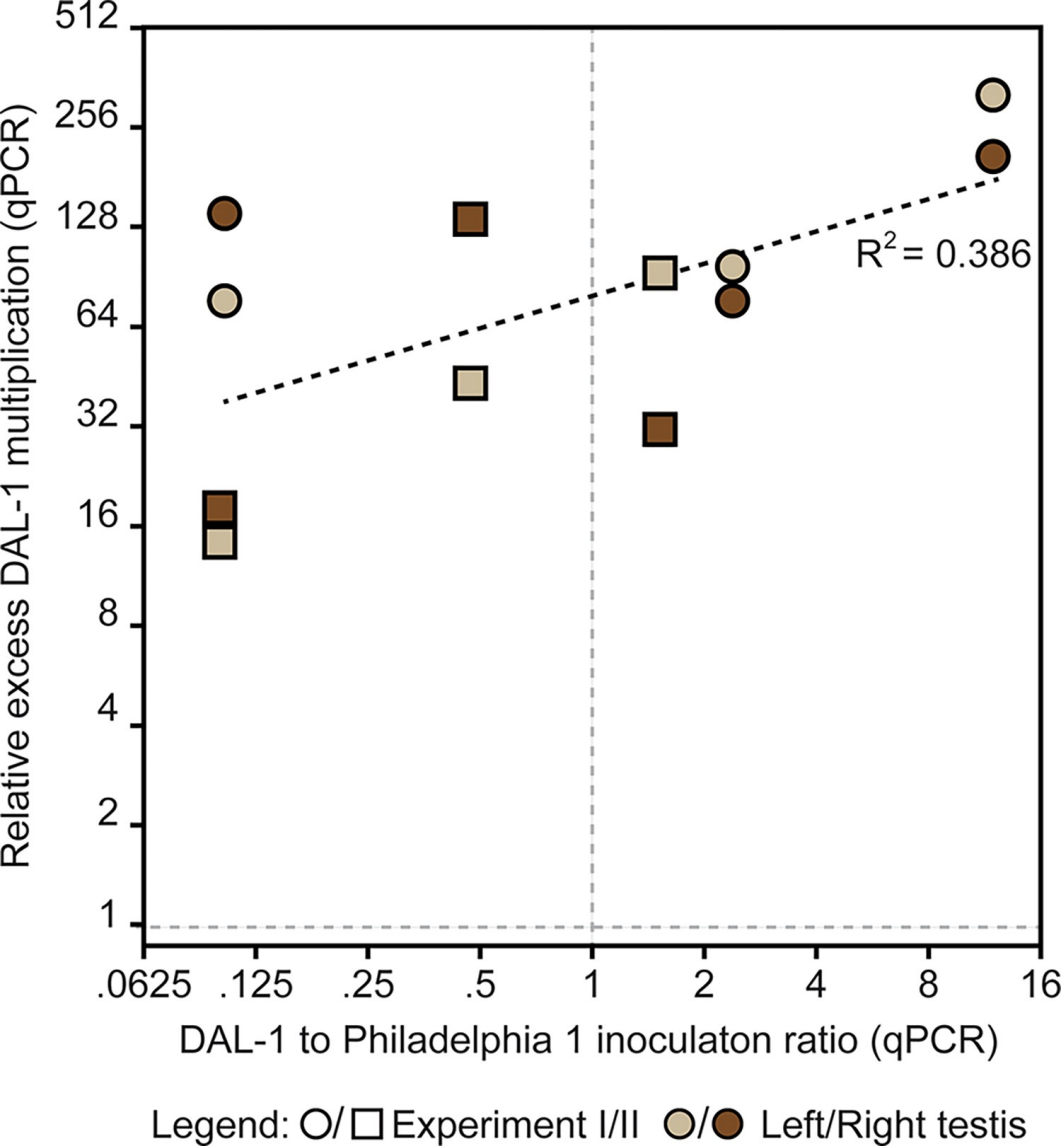
Multiplication of TPA strains during intratesticular co-infection in rabbits. Results from two independent experiments using different TPA inoculation ratios (n = 6; four control rabbits infected with only one TPA strain are not shown). DAL-1 overgrew Philadelphia 1 in all co-infections (n = 12 testes). Relative excess of DAL-1 multiplication (increase in the ratio between DAL-1 and Philadelphia 1 during co-infection) depended on the inoculation ratio as indicated by the dotted black line. TPA numbers were quantified in each testis using DAL/Phi discriminative qPCR. The relative excess of DAL-1 multiplication obtained from the left (dark brown) and right (light brown) testis is shown for each experimental rabbit. Please note that the x-axis reflects the inoculation ratio calculated from qPCR values instead of DFM-estimated ratios. Complete qPCR results are shown in S2 Table.

To overcome the individual variability between rabbit passages, rabbits were simultaneously infected with both TPA strains. Moreover, different DAL-1 to Philadelphia 1 ratios were prepared to assess if potential differences in the growth rate reflect competition for the same nutrients. The intratesticular co-cultivation of treponemes was performed in two independent experiments (I and II); the experimental scheme is shown in Fig 1. The inoculation dose was calculated using DFM to minimize the time needed to prepare treponemal mixtures, and later, it was more precisely determined by qPCR. In fact, qPCR quantification confirmed inoculation ratios estimated from DFM (S2 Table). The more exact qPCR ratios from both inoculum and testes sampled at the end of the experiment (14 days) were used to calculate treponemal multiplication. The results showed that the DAL-1 strain overgrew Philadelphia 1 in all cases of co-infection, regardless of the inoculation ratio (Fig 4) and the significantly different inoculation doses of TPA strains used in two experiments (S2 Table). The ratio of DAL-1 multiplication compared to Philadelphia 1 multiplication (relative excess DAL-1 during multiplication) was calculated and the median value was found to be 84.85× (119.05× and 37.5× in Experiment I and II, respectively; for values in individual testes, see S2 Table), showing that DAL-1 multiplied faster *in vivo* than Philadelphia 1.

The relative excess DAL-1 multiplication appears to be higher with increased initial inoculation ratios, although this correlation was weak (R^2^ = 0.386) (Fig 4).

## Discussion

In this study, we compared the growth characteristics of TPA DAL-1 and Philadelphia 1 strains *in vitro* and during experimental intratesticular infections of laboratory rabbits. Under both conditions, the DAL-1 grew significantly faster than the Philadelphia 1 strain. Standardization of *in vitro* experiments was easily achievable because all the components of the *in vitro* cultures and incubation conditions were the same for both analyzed TPA strains. However, standardization of experimental rabbit infection can be challenging since it requires many animals, and the outcomes of individual passages are highly variable (Fig 3). An intratesticular co-infection scheme was used to eliminate factors causing variability, such as differences in rabbit genotype, age, behavior, and the precise location of inoculations. Moreover, based on sequence differences in the TP0136 gene between both TPA strains, a new strain-specific qPCR protocol was designed to detect each strain separately in intratesticular co-infection. To minimize differences in the detection procedure, both qPCR protocols used the same FAM probe, one identical primer for the TP0136 gene, and amplified similar amplicons.

Compared to Philadelphia 1, the DAL-1 grew about 1.5 times faster under *in vitro* conditions, as revealed by 7-day-long cultivation and qPCR evaluation (Fig 2). This is additional evidence that strains in the SS14-like group have longer *in vitro* generation times than the Nichols-like strains [11]. Experimental rabbit infection by DAL-1 resulted in clinical manifestation about a week sooner than Philadelphia 1 (i.e., two vs. three weeks). Given that the onset of symptoms is a function of the TPA numbers and the used inoculation numbers were similar for both TPA strains, it can be inferred that DAL-1 has shorter *in vivo* generation time. Using identical *in vivo* conditions during co-infections, the DAL-1 strain outgrew the Philadelphia 1 strain in both testes of all six infected rabbits (i.e., all together in 12 biological experiments of co-infection; Fig 4). During the two-week-long co-infection experiment, the DAL-1 strain overgrew Philadelphia 1 by a median factor of 84.85. In addition, the initial ratio between DAL-1 and Philadelphia 1 in the co-infection experiments appeared to slightly modulate the differences in growth rates (Fig 4). Inoculations with higher DAL-1 to Philadelphia 1 ratios showed higher relative DAL-1 multiplication than inoculations with lower DAL-1 to Philadelphia 1 ratios. This finding suggests that the ability of the testis to support the growth of treponemes is limited and that direct competition between strains for available nutrients takes place during co-infection. The competition between TPA strains *in vivo* is also suggested by a higher excess of DAL-1 multiplication during co-infection compared to *in vitro* results (84.85 vs. 2.30 after 14 days, respectively).

Both TPA strains used in this study were passaged for a limited time in rabbits after isolation from humans [12, 14] and are generally not considered adapted to rabbits. The second co-cultivation experiment was preceded by several individual serial passages of both TPA strains to minimize potential differences related to adaptation to rabbit infection. Genetic adaptation of TPA strains to rabbit infection during long-term propagations in rabbits is widely speculated, but there is no clear genetic evidence of this process. While Giacani et al. [20] suggested the presence of such mutations by genetic comparisons of the Nichols strain to other Nichols-like strains and Edmondson et al. [21] demonstrated the microevolution of the Nichols strain, Grillová et al. [22] tested the DAL-1 strain for genome stability and adaptation in continual rabbit cultivation representing more than 100 TPA generations (i.e., 142 days) during which the allelic profile of DAL-1 remained stable. Moreover, the genetic adaptation to rabbits appears to be a relatively long process corresponding to the relatively low estimated mutation rates of pathogenic *T*. *pallidum*, where it took over a decade to fix a single mutation [23, 24].

In general, the longer generation time of Philadelphia 1 compared to the DAL-1 suggests important physiological differences between the two tested TPA strains, including their pathophysiology. On the other hand, several other possibilities could explain the *in vivo* growth rate differences, including more efficient removal of Philadelphia 1 by the host immune system, increased dissemination of Philadelphia 1, and/or higher/different nutrient requirements of Philadelphia 1 compared to DAL-1. Since the growth rate differences were also observed under *in vitro* conditions, the first explanation appears to be more convincing.

This study has several limitations, including the use of only two TPA strains due to difficulties in maintaining viable TPA cultures under laboratory conditions and the time-consuming and expensive nature of rabbit experiments. Another limitation was single sampling after the experiment, i.e., without several sampling points in between, which would have allowed a more dynamic interpretation of results but would have required more experimental animals. The number of treponemes in the inoculation suspensions can be inaccurate when assessed using DFM, especially in cases with less than one treponeme per field. This limitation was overcome by a more precise assessment of the numbers of treponemes in the inoculation suspensions using qPCR.

While the majority (> 70%) of recent human infections have been caused by SS14-like strains (including Philadelphia 1) [9, 16, 25–27], about half of the propagated laboratory strains belong to the Nichols-like group (including DAL-1) [8, 9]. Both Nichols-like and SS14-like isolates differ in the genetic diversity within each group [15], the prevalence of macrolide resistance [24], the presence of multiple primary ulcers in infected patients [28], and PCR positivity during secondary syphilis [24], suggesting that there are important yet unrevealed genetic and physiological differences between these groups of TPA strains. Our experiments showed important growth differences between TPA strains in rabbit cell-supported cultivation and in rabbit experimental infection, suggesting that Nichols-like strains may be better adapted for rabbit infections and explaining their preponderance among laboratory rabbit-propagated TPA strains. However, it is unclear if the observed differences also apply to other TPA strains of the Nichols-like and SS14-like groups, including contemporary TPA strains, and if there are similar differences between them during human infection. Further studies will be needed to answer these questions.

## Supporting information

S1 Table Longitudinal propagation of TPA strains—details on individual rabbit infections. (XLSX)

S2 Table Intratesticular co-infection of TPA strains—qPCR results. (XLSX)

S3 Table Specificity of TP0136 qPCR detection and comparison of TP0136 and TP0105 qPCR detections. (XLSX)

S4 Table *In vitro* cultivation of TPA strains–DFM and qPCR results. (XLSX)
